# SNES: single nucleus exome sequencing

**DOI:** 10.1186/s13059-015-0616-2

**Published:** 2015-03-25

**Authors:** Marco L Leung, Yong Wang, Jill Waters, Nicholas E Navin

**Affiliations:** Department of Genetics, MD Anderson Cancer Center, Houston, TX USA; Department of Bioinformatics and Computational Biology, MD Anderson Cancer Center, Houston, TX USA; Graduate Program in Genes and Development, Graduate School of Biomedical Sciences, University of Texas Health Science Center at Houston, Houston, TX USA

## Abstract

Single-cell genome sequencing methods are challenged by poor physical coverage and high error rates, making it difficult to distinguish real biological variants from technical artifacts. To address this problem, we developed a method called SNES that combines flow-sorting of single G1/0 or G2/M nuclei, time-limited multiple-displacement-amplification, exome capture, and next-generation sequencing to generate high coverage (96%) data from single human cells. We validated our method in a fibroblast cell line, and show low allelic dropout and false-positive error rates, resulting in high detection efficiencies for single nucleotide variants (92%) and indels (85%) in single cells.

**Electronic supplementary material:**

The online version of this article (doi:10.1186/s13059-015-0616-2) contains supplementary material, which is available to authorized users.

## Background

Single-cell sequencing methods have the potential to provide great insight into the genomes of rare subpopulations and complex admixtures of cells, but are currently challenged by extensive technical errors and poor physical coverage data. While much progress has been made in developing single-cell RNA sequencing methods [1-4], the development of genome-wide DNA sequencing methods has proven to be more challenging [5,6], owing to the fact that single cells contain thousands of copies of each mRNA molecule, but only two copies of each chromosome. Therefore each cell provides only two template DNA molecules for whole-genome-amplification (WGA) reactions and errors that occur in the initial rounds of amplification are inherited by all subsequent molecules. In our previous work we developed the first single-cell genome sequencing method, Single-Nucleus-Sequencing (SNS), which utilized DOP-PCR to generate about 10% coverage breadth of an individual cell [7,8]. Coverage breadth is defined as the percentage of nucleotide sites in the single-cell data with ≥1X coverage depth. However, while SNS was adequate for copy number detection using large genomic intervals (54 kb), it could not detect mutations at base-pair resolution. Two subsequent methods were developed that use multiple-displacement-amplification (MDA) [9] and multiple-annealing-looping-based-amplification-cycles (MALBAC) [10] to increase coverage breadth during WGA. While pioneering, these studies increased coverage breadth at the cost of introducing high false positive and false negative error rates, due to excessive over-amplification (1:1e6) of the DNA from a single cell from 6 picograms to microgram concentrations. Consequently, it was necessary to call variants across most of the single cells to reduce the high false positive (FP) technical errors, which is equivalent to sequencing the bulk tissue *en masse*.

To mitigate technical errors, we recently developed a method called Nuc-Seq, which utilizes G2/M cells to perform single-cell genome sequencing [11]. While this approach was suitable for analyzing highly proliferative cells, such as cancer cells, it was not suitable for the analysis of normal cells or slowly dividing populations. To address this problem, we developed a new approach called single nucleus exome sequencing (SNES) that builds upon our previous method. SNES combines flow-sorting, time-limited isothermal multiple-displacement amplification (MDA), exome capture, and next-generation sequencing (NGS) to generate high coverage (96%) data for the accurate detection of point mutations and indels in single mammalian cells. SNES has several improvements over Nuc-Seq, including: (1) improved exome capture performance; (2) time-limited isothermal amplification; (3) enhanced MDA polymerases; (4) efficient DNA ligases; (5) quality control (QC) of WGA using qPCR panels; and (6) cost reduction by using standard reagents instead of commercial WGA kits. Importantly we show that SNES can be applied to either G1/0 or G/2 M cells, opening up new avenues of investigation into single-cell genomics studies of normal tissues and slowly proliferating cells (for example, stem cell or cancer stem cells).

## Results and discussion

### Experimental approach and quality control assays

**Figure 1:**
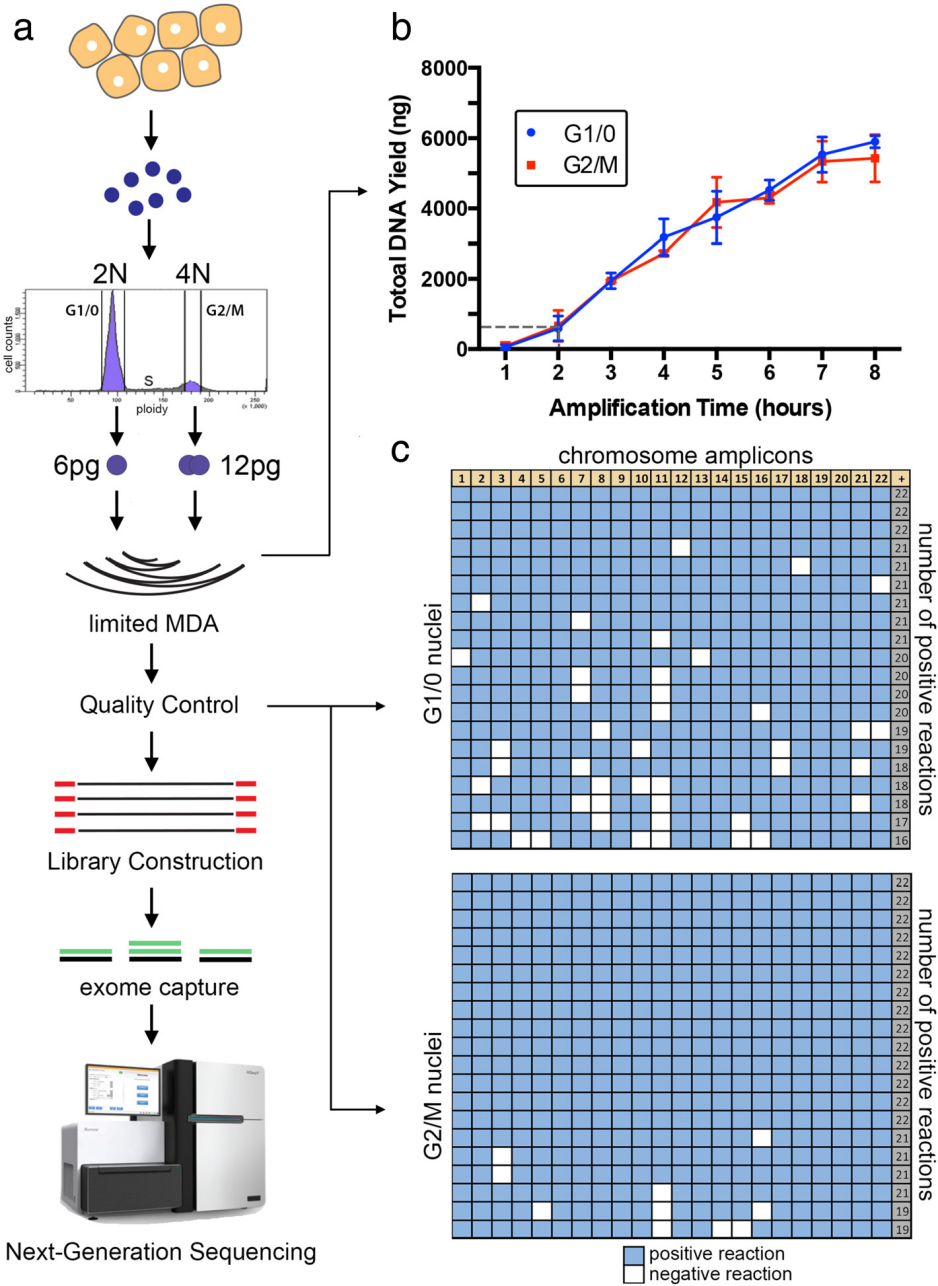
**SNES method and WGA quality control. (a)** Nuclear suspensions were prepared from tissues, stained with DAPI and flow-sorted. Single nuclei were isolated by gating the G1/0 or G2/M ploidy distributions and deposited nuclei singly into a 96-well plate. Multiple-displacement-amplification is performed using Φ29 to perform WGA. **(b)** Time-course of WGA showing total DNA yield from single nuclei. **(c)** Quality control assay using a panel of 22 chromosome-specific qPCR primers to determine the WGA amplification efficiency of each single nucleus.

To perform SNES nuclear suspensions are prepared from fresh or frozen tissue using a DAPI-NST lysis buffer (Figure 1a). Single nuclei are flow-sorted into individual wells by gating distributions of ploidy at 2 N (G1/0) or 4 N (G2/M). Alternatively, this approach can be applied to gate G1/0 or G2/M cells from aneuploid tumors, which also have G2/M distributions at higher ploidy indexes (Additional file 1: Figure S1). Single nuclei are then deposited into individual wells of a 96-well plate containing nuclear lysis buffer. The 6 picograms (2 N) or 12 picograms (4 N) of gDNA from each nucleus is incubated with the Φ29 polymerase (New England Biolabs) and modified random hexamer primers to perform time-limited MDA. To determine the optimal isothermal timeframe, we performed time-series MDA reactions using G1/0 and G2/M cells over 8 h (Figure 1b). From this curve, we determined 120 min to be the minimum time-frame required to generate approximately 500 ng of DNA from a single cell, providing sufficient input material for constructing libraries, exome capture, and performing the necessary quality control assays.

To evaluate WGA efficiency we performed qPCR on each single nucleus WGA reaction using a set of 22 primer pairs that target each chromosome independently (Additional file 2: Table S1, methods). Single nuclei with 22/22 amplicons were selected for subsequent library construction and next-generation sequencing. Our data showed that G2/M cells resulted in an improvement over G1/0 cells for WGA efficiency, with 70% (14/20) single cells having the full set of chromosomes amplified in G2/M cells compared to 15% (3/20) in G1/0 cells (Figure 1c). Additional G1/0 WGA experiments and QC were subsequently performed to obtain nine SKN2 cells in total for the single-cell sequencing experiments.

Notably, performing QC analysis with qPCR on cancer cells may lead to diminished signal due to hemizygous deletions, and occasionally rare focal homozygous deletions [12,13]. Therefore users may want to consider looser filtering criteria (20/22 amplicons). However this has not been a problem in our previous work, in which we performed QC on single breast tumor cells using standard PCR and found that >20 amplicons were detected in 80 single cells [11]. Single cells that passed QC for WGA were used to construct sequencing libraries using a low-input TA cloning protocol starting with 100 ng of input material (see Methods). During library construction a unique 6 bp barcode was added to each single-cell library for sample multiplexing. We pooled four single-cell libraries together into one reaction for exome capture (TruSeq, Illumina) and next-generation paired-end sequencing on the HiSeq2000 system (Illumina) using 100 paired-end cycles. The number of cells that can be multiplexed is a function of the amount of data that is generated from the sequencing platform and the size of the exome target region.

### Measuring coverage performance and uniformity

**Figure 2:**
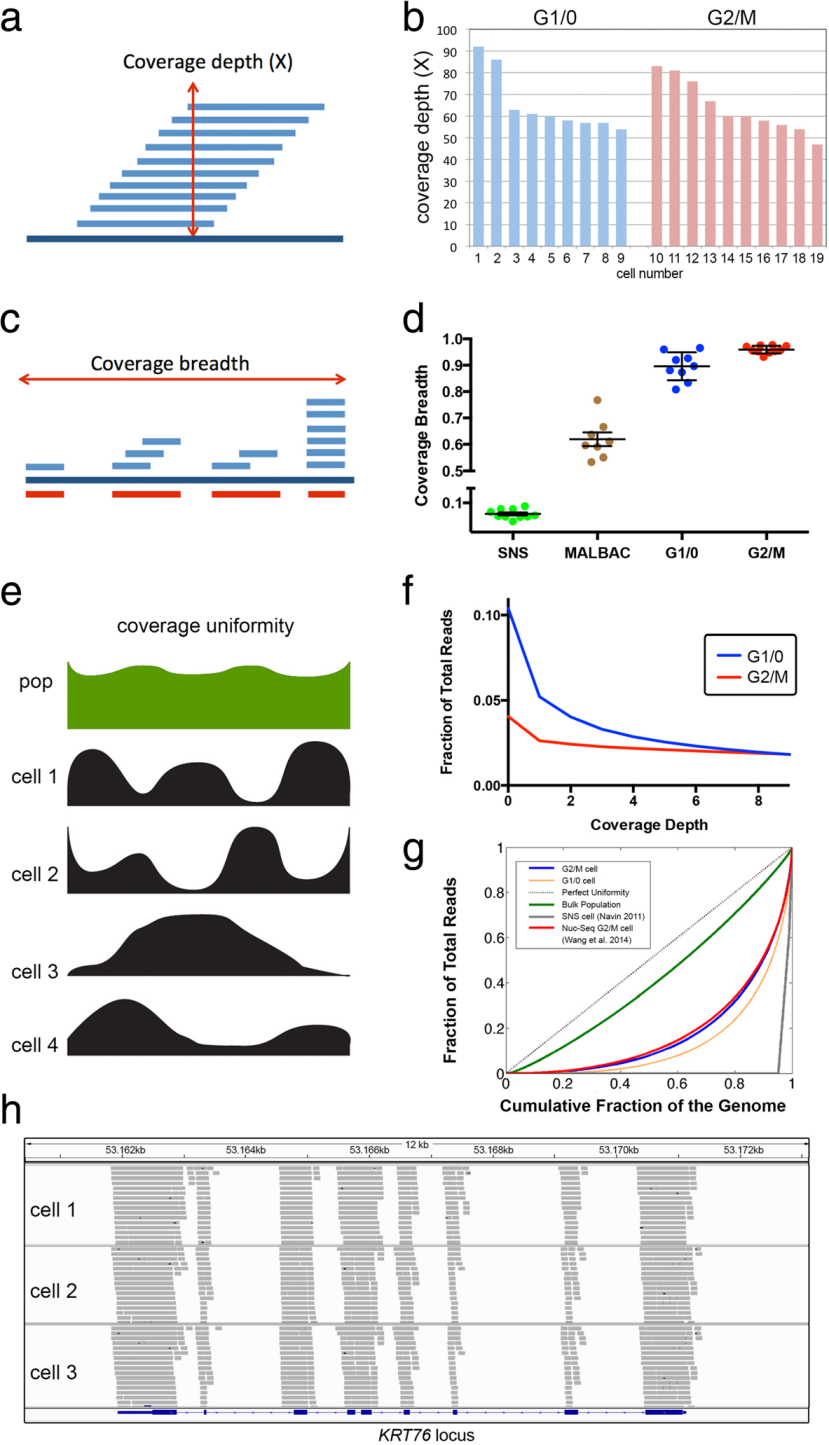
**Coverage performance and metrics. (a)** Coverage depth diagram. **(b)** Coverage depth data for G1/0 and G2/M single cells. **(c)** Coverage breadth diagram. **(d)** Coverage breadth data for exome region of G1/0 and G2/M single cells compared to previous studies using SNS [7] and MALBAC (Ni *et al.* [28]). Error bars show SEM. **(e)** Coverage uniformity diagram. **(f)** Coverage depth distribution for sites with low coverage in G1/0 and G2/M single cells. **(g)** Lorenz curves of coverage uniformity, showing values for perfect coverage, millions of SKN2 reference cells, Nuc-Seq single cell [11], single cells from G1/0 and G2/M distributions, and SNS cell [7]. **(h)** Capture performance of sequence reads across exons in the *KRT76* locus for three single cells.

To determine the coverage performance and error rates of SNES, we used a normal isogenic female fibroblast cell line (SKN2), in which we assume that the variants present in a single cell will be highly similar to the reference population sample. Any deviations from the reference variants were considered to be technical errors, and were used to calculate the error rates (see Methods). We sequenced the population of cells at high coverage depth (59X) and breadth (99.76%) to obtain a reference set of whole-genome variants. We then applied SNES to sequence nine single cells that were gated from the G1/0 stage of the cell cycle and 10 single cells from the G2/M stage (Additional file 3: Table S2). We aligned the single-cell data to the human genome using our processing pipeline and eliminated sequence reads with multiple mappings and PCR duplicates (see Methods). As expected, all of the single cells showed very similar coverage depth distributions, irrespective of whether they were gated from the G1/0 or G2/M distributions (*P* = 0.85, *t*-test), which is important for the subsequent comparisons (Figure 2a and b).

In order to assess coverage performance, we calculated coverage breadth (sites with ≥1X coverage) (Figure 2c) and coverage uniformity (evenness) (Figure 2e). Our data suggest that coverage breadth (≥1X) significantly (*P* = 0.0021, *t*-test) increased in the G2/M cells (95.94%, ± 0.005 SEM) relative to the G1/0 cells (89.60% ± 0.018 SEM) (Figure 2d). This results in the number of site with sufficient coverage depth for variant calling at 73.54% in G1/0 cells compared to 84.34% in G2/M cells. To assess coverage uniformity, we plotted the fraction of the exome covered as a function of coverage depth (Figure 2f). These plots show that the G2/M cells achieved more even coverage uniformity at sites with low coverage depth compared to the G1/0 cells. To further investigate coverage uniformity, we calculated Lorenz curves and plotted data for perfect uniformity, a genomic DNA population sample and mean data for the G1/0 and G2/M single cells, as well as data from our previous Single-Nucleus-Sequencing method (Figure 2g) [14]. These curves show a large improvement in coverage uniformity using G2/M cells compared to the G1/0 cells, and both showed vast improvements over our previous SNS approach [7]. We also calculated the on-target performance for data in the exome region of single cells, and found very high percentages (mean = 67.33%) for G1/0 and G2/M cells (Figure 2h), which is equivalent to previous reports (55% to 85%) of exome capture efficiencies using millions of cells [15].

### Estimating technical error rates

**Figure 3:**
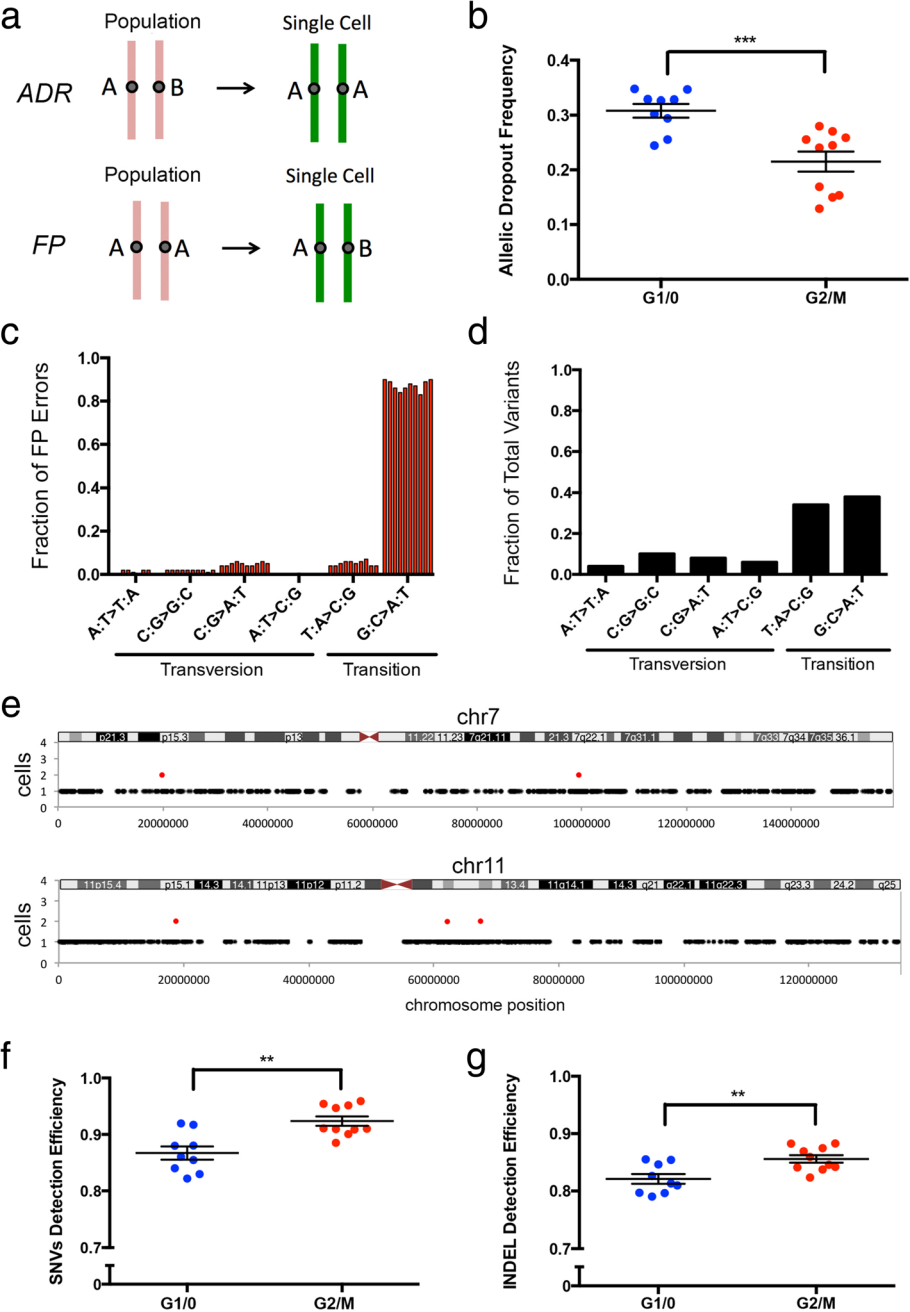
**Error rates and detection efficiencies. (a)** Illustration of technical errors, including the allelic-dropout rate (ADR) and false positive (FP) error rate. **(b)** Allelic dropout rates for single-cell experiments using G1/0 and G2/M single cells. **(c)** Spectrum of FP errors detected in the G2/M single-cell data, with each column representing a single cell. **(d)** Spectrum of single nucleotide variants detected in the SKN2 population data. **(e)** Distribution of FP errors on chromosome 7 and 11 that occur in single cells (black) or in two or more cells (red). **(f)** Detection efficiency for single-nucleotide variants in the G1/0 cells and G2/M cells calculated from the exome sequencing data. **(g)** Detection efficiency for indels in the G1/0 cells and G2/M cells calculated from single-cell exome sequencing data. Error bars in all panels represent the SEM.

To calculate the technical error rates we filtered the reads by mapping quality, base quality, and clustered regions [16]. We then performed local realignment around indels (see Methods). From these data we identified single-nucleotide variants (SNVs) and indels using the Unified Genotyper (GATK), following our processing pipeline (Additional file 4: Figure S2) [17]. Major sources of technical errors that occur during WGA include the allelic dropout rate (ADR) and the FP error rates (Figure 3a) [18,19]. Previous studies have reported very high (43.09%) allelic dropout rates in single-cell exome sequencing data [19,20]. In comparison, our data show that SNES significantly (*P* = 7e-4, *t*-test) reduced the allelic dropout rates to 30.82% (±0.013, SEM) in G1/0 cells and 21.52% (±0.019, SEM) in G2/M cells (Figure 3b). These calculations are based on sites in which both the single cells and population sample have sufficient (≥6X) coverage depth (in order to eliminate sites with low coverage in which WGA did not necessarily lead to allelic dropout). An alternative approach for calculating the ADO includes all heterozygous sites in the population and single cell sites regardless of coverage depth, which results in an ADR of 43.84% for G1/0 cells and 27.21% for G2/M cells.

Next, we calculated the FP error rate, which is caused by the infidelity of the Φ29 polymerase (error rate = 1e-7) during isothermal amplification [21]. From our data we calculated a FP error rate of 3.2e-5 for SNVs, which is equivalent to 32 errors per megabase. This FP error rate is higher than our previous estimates for whole-genome single-cell sequencing with Nuc-Seq, but can be explained by the increased isothermal WGA timeframe and additional PCR cycles required to generate sufficient DNA for exome capture and enrichment. We investigated the spectrum of the FP errors and found that 82.3% were C > T and G > A transitions (Figure 3c), showing a significant bias relative to the normal transition and transversion spectrum in the population of fibroblast cells (Figure 3d). Importantly, we found that the majority of the FP errors occurred at random sites in the genomes of single cells, with few mutations occurring at recurrent sites in two or more cells (Figure 3e, Additional file 5: Figure S3). This distribution allows the FP error rates to be mitigated by calling mutations in two (FP: 3.2e-5^2^ = 1.02e-9) or more (FP: 3.2e-5^n^) single cells. Using two or more cells in variant calling is possible in most single-cell studies, which normally seek to analyze large numbers of cells.

We also investigated the distribution of allelic dropout events in the single-cell data. By comparing the allelic dropout events from both alleles, our data showed that there is a slight bias towards AB → BB dropout events, when compared to AB → AA events in both the G1/0 and G2/M cells (Additional file 6: Figure S4). We hypothesize that this bias is likely due to mismatch hybridization inefficiency of the exome capture probes to the B alleles, since they were designed for the A allele sequence (reference human genome assembly). Next we examined the distribution and recurrence of allelic dropout events by examining their frequency across multiple single cells (Additional file 7: Figure S5). Our data show that in contrast to the random distribution of FP errors that occur at different site in single cells, allelic dropout errors sometimes occurred at recurrent positions in multiple single cells (Additional file 7: Figure S5). On average we observed that 2.55 cells out of 19 single cells shared a recurrent allelic dropout event at the same nucleotide position. These regions are important to note in single-cell studies and should be filtered, since they can be misinterpreted as biological variation in SNV prevalence, when in fact they are likely to be technical errors.

### Measuring detection efficiencies

We calculated the detection efficiencies, to measure the proportion of the SNVs and indels that were successfully detected in each single fibroblast cell exome. For SNVs we detected 92.37% (±0.008, SEM) of the variants in the single cells (mean = 32,369/34,982) in the G2/M cells, and 86.71% (±0.012, SEM) in the G1/0 cells (mean = 25,753/29,549) (Figure 3f). In comparison, previous studies using MALBAC [9] reported detection efficiencies of only 76% for SNVs. An alternative approach is to calculate the SNV detection efficiency at all variant sites in the reference, regardless of the coverage depth in the single-cell and population sample. This calculation results in a detection efficiency for SNVs of 60.64% for G1/0 cells and 76.22% for G2/M cells. We also calculated the detection efficiency for indels, which is 85.60% (±0.007 SEM) for G2/M cells (mean = 2,448/2,856), and 82.11% (±0.009 SEM) for G1/0 cells (mean = 1,926/2,336) (Figure 3g). To our knowledge, this is the first report showing that indels can accurately be detected in the genomes of single mammalian cells.

## Conclusions

We report the development of a novel single-cell exome sequencing method called SNES that can achieve high coverage (96%) data from the exome of a single mammalian cell. From these data we show that we can accurately detect SNVs and indels at base-pair resolution. The technical performance in coverage improvement is due to multiple factors, including an improved Phi29 polymerase (New England Biolabs), time-limited isothermal amplification and the use of a 22-chromosome qPCR panel to eliminate cells with poor WGA performance prior to exome capture and sequencing. In contrast to our previous method [11], SNES eliminates the requirement for Tn5 transposases for library construction, which can introduce integration biases in the human genome and lead to coverage non-uniformity [22-25]. By performing time-limited isothermal MDA with an improved Phi29 polymerase (New England Biolabs) we were able to mitigate FP and FN error rates, resulting in improved detection efficiencies for SNVs and indels. Importantly, the SNES protocol eliminates commercial kits for cell isolation, WGA, and library construction, thereby reducing the cost of generating a single-cell library to approximately $30 per cell (not including the exome capture reagents and sequencing costs). This will enable a large number of cells to be analyzed and multiplexed, which is the goal of most single-cell sequencing studies.

In our study, we performed a direct comparison of data derived from G1/0 and G2/M single cells, which shows that both cells performed well for coverage uniformity and breadth. However our data show that using G2/M cells will lead to even further technical improvements in the allelic dropout rates. In the future, further technical improvements may be achieved by combining SNES with microfluidic platforms (for example, Fluidigm) which have been shown to decrease errors when nanoliter volumes are used for MDA reactions [5,26,27]. In contrast to most single cell sequencing methods to date [5,9,10,27] SNES utilizes nuclei instead of cells for analysis. Nuclei have several advantages over using cells: (1) nuclei can be stained with DAPI and gated to avoid collecting cells that are degraded, apoptotic, or replicating; 2) nuclei can be deposited more accurately to avoid sequencing multiple cells; and (3) nuclei can be isolated from archival frozen tissue samples, that have been stored for decades [5,10,20,28]. The last point is crucial for single-cell sequencing of archival tissues, because freezing ruptures the cytoplasmic membrane of most cells, but leaves the nuclear membrane intact from which nuclear suspensions can easily be prepared. However, nuclei also have several notable limitations, including potentially missing DNA from micronuclei [29] and the fact that cell surface markers cannot be used to isolate specific populations (for example, by gating during flow-sorting). Thus the choice of using nuclei vs. cells will depend largely on the specific requirements of the research project. In closing, we expect that SNES will have broad applications in many diverse fields of biology, including cancer research, microbiology, neurobiology, development, and prenatal genetic diagnosis, and will lead to vast improvements in our fundamental understanding of human diseases.

## Methods

### Cell line sample

SKN2 is an isogenic human fibroblast cell line that was obtained from the Cold Spring Harbor Laboratory (Dr. Michael Wigler). SKN2 was cultured using Dulbecco’s Modified Eagle Medium with 10% fetal bovine serum, penicillin/streptomycin and L-glutamine.

### SNES experimental protocol

#### Isolating single nuclei by flow-sorting

Nuclei were isolated from the fibroblasts using an NST-DAPI buffer (800 mL of NST (146 mM NaCl, 10 mM Tris base at pH 7.8, 1 mM CaCl_2_, 21 mM MgCl_2_, 0.05% BSA, 0.2% Nonidet P-40)), 200 mL of 106 mM MgCl2, 10 mg of DAPI, and 0.1% DNase-free RNase A. Cells were trypsinized and lysed using the NST-DAPI buffer. The nuclear suspension was filtered through 37-μm plastic mesh prior to flow-sorting. Single nuclei were sorted using the MoFlo Astrios Cell Sorter (Beckman) by gating cellular distributions with differences in their total genomic DNA content according to DAPI intensity. Single nuclei were sorted into individual wells in a 96-well plate. Each well was preloaded with 3.5 μL of lysis buffer (1 M DTT, 100 mM sodium phosphate, 10 mM Tris pH 8.0, and 6 M guanidine hydrochloride and PBS). After flow-sorting, the plate was centrifuged at 700 rpm for 1 min and incubated at 65°C for 10 min. After adding 1.5 μL of neutralization buffer (800 mM Trizma hydrochloride) to each well, the volume was 5 μL.

#### Whole-genome amplification by time-limited multiple-displacement amplification

Whole-genome amplification was performed on single flow-sorted nuclei using 10 units of Φ29 polymerase and 10× Φ29 buffer (NEB cat#M0269L), 1 mM dNTP (GE Healthcare, cat#28-4065-51), and 50 μM random hexamer (phosphorothioate modification on the two 3’-terminal nucleotide - NNNN*N*N - synthesized by Sigma Aldrich) to each well. The total 50 μL reactions were mixed by gently pipetting up and down and spinning down the reaction. Reactions were incubated at 30°C for 120 min to obtain approximately 500 ng of DNA. The polymerase was denatured subsequently at 65°C for 3 min. The amplified DNA was purified using DNA Clean & Concentrator-5 columns (Zymo D4004).

#### WGA quality control using qPCR chromosome panels

To evaluate the WGA amplification efficiency of each single-cell reaction we designed 22 pairs of primers (Sigma Aldrich) to target 22 loci on different chromosomes for qPCR (Additional file 2: Table S1). The primer sequences are listed below. For each qPCR reaction 5 ng of DNA was used for the KAPA Taq PCR kit (Kapa #BK1001). The qPCR reactions were run on the ABI 7500 system (Applied Biosystems) in 96-well plates. The qPCR conditions used were: 95°C for 3 min, followed by 45 cycles (95°C for 20 s, and 60°C for 30 s). Single cell WGA reactions that show positive qPCR reactions are selected for subsequent library construction and next-generation sequencing.

#### Construction of barcoded sequencing libraries

WGA reactions that passed QC were sonicated at 350 bp using Covaris S220. We used 100 ng of DNA to construct sequencing libraries by TA cloning using KAPA Library preparation kit (Kapa Biosystems, cat#KK8232), in accordance with the manufacturer’s protocol. Libraries were quantified by real-time qPCR using Library Quantification Kit (Kapa Biosystems, cat# KK4835). We used the TruSeq Exome Enrichment Kit (Illumina cat# FC-121-1008) for exome capture in accordance with the manufacturer’s protocol using eight cycles of PCR enrichment. Final concentrations were measured prior to sequencing by qPCR using the Kappa Library Quantification Kit. Libraries were sequenced using 100 cycle paired-end flow-cell lanes on the HiSeq2000 system (Illumina, Inc.) for 100 cycles. Data were processed using CASAVA 1.8.1 pipeline (Illumina, Inc) converting BCL basecall files to fastq files.

### Data processing and analysis pipeline

Sequence reads in FASTQ files were aligned to the human genome (hg19) using the Bowtie 2 alignment software [30]. Samtools (0.1.16) was used to convert SAM files to compressed BAM files and sort BAM files by coordinate [31]. The Genome Analysis Toolkit (GATK v1.4-37) was used to locally realign the BAM files at intervals with indel alignment errors [17]. To eliminate PCR duplicates, we removed sequences with identical start and end coordinates using Picard software [32]. Reads with mapping quality MQ <40 were filtered from the BAM files. We used GATK Unified Genotyper to detect single nucleotide variants (SNVs). All single cells and reference samples were processed together to generate a single VCF4 file. We required a minimum base quality (mbq) of 20 for the base to be considered during variant detection. Coverage depth at a given locus of greater than 2,500 reads was down sampled to expedite analysis processing. We used the GATK variant recalibrator to filter the output at default sensitivity level. A minimum coverage depth of 6 and at least 2 reads with variant allele was used for further filtering of SNVs. SNVs in clustered regions with neighboring SNVs within 10 bp were filtered from the data to remove FPs. We then used GATK SelectVariants to separate SNVs into VCF4 files for downstream analysis. The processing pipeline is outlined in Additional file 4: Figure S2.

### Calculation of coverage metrics

Reads with multiple mappings in the human genome were filtered from the BAM files. Sequence reads with unique mappings were used for calculating coverage metrics. Coverage depth and breadth were calculated using BEDTools by running genomecoverageBED using the BED file from the TruSeq capture region of 62,286,318 bp [33]. Coverage breadth is defined as the percentage of the exome with at least 1× read coverage, while coverage depth refers to the mean number of read counts across all the bases of a sequenced sample. Lorenz curves [14] were calculated to determine coverage uniformity in the single cell and population samples. Briefly, sequence reads were aligned with bowtie2 using unique mappings and PCR duplicates were removed with Picard. From the BAM files we ran samtools mpileup with the following parameters: ‘-A -B -d1000000000’ to determine the read counts for every base in the human genome reference assembly HG18. The depth values were sorted using Unix sort with ‘-n’ parameter and a custom perl script was used to read the sorted depth values and calculate the cumulative fraction of the genome that was covered and the cumulative fraction of reads. The curves for each cells and population samples were plotted in Matlab (Mathworks).

### Calculation of technical error rates

The allelic dropout rate (ADR) is defined as the mean fraction of homozygous sites in the single-cell samples (*Hom*_s_) where the population reference sample is heterozygous (*Het*_p_) at the same nucleotide site. These calculations were made using all of the G1/0 or all of the G2/M single-cell fibroblast exome sequencing data independently. In these calculations both sites (reference and single cell) required a minimum of 6X coverage depth to call variants.

$$ \boldsymbol{A}\boldsymbol{D}\boldsymbol{R} = \frac{1\ }{n}\ {\displaystyle \sum_{i=1}^n}\ \frac{Ho{m}_s}{He{t}_p} $$

The false positive rate (FPR) is defined as the number of heterozygous sites in the single cell sample (*Het*_s_) divided by the number of sites in the population reference sample (*Hom*_p_) that are homozygous for the reference allele at the same nucleotide site.

$$ \boldsymbol{F}\boldsymbol{P}\boldsymbol{R}=\frac{1}{n}\ {\displaystyle \sum_{i=1}^n}\ \frac{He{t}_s}{Ho{m}_p} $$

### Calculation of detection efficiencies

The detection efficiencies are calculated from the VCF4 variant files after the filtering steps have been performed. The filtered multi-VCF4 file is partitioned into separate files for SNVs and indels. For each line in the VCF file we add a binary string indicating the absence or presence of each variant in the single-cell samples or the reference population sample. For each variant site in the population sample, we identify variant sites in the single-cell samples with sufficient coverage depth (≥6X). From the binary string we determine if the variant is present/absent in each single cell relative to the population reference sample. We define a variant as being detected if the reference allele is AB and the single-cell data are either AB or BB. The mean detection efficiencies for indels and SNVs are then computed across all of the single cells.

### Data access

The data from this study has been deposited into the Sequence Read Archive (SRA) under accession SRP046355.

## Additional files

Additional file 1: Figure S1. G1/0 and G2/M ploidy distributions in aneuploid breast tumors. Nuclei were prepared from frozen breast tumors and stained with DAPI. The nuclear suspensions were analyzed by cytometric analysis showing the distributions of total DNA content. The aneuploid distributions are highlighted for G1/0 and the corresponding G2/M populations in each frozen tumor sample. Additional file 2: Table S1. Chromosome specific qPCR primers. Summary table of the chromosome-specific primer panel DNA sequences that are used to perform quality control of the single cell WGA reactions. Additional file 3: Table S2. Single nuclei exome sequencing metrics. Summary table for the coverage and sequencing metrics from single-cell exome sequencing of the G1/0 and G2/M fibroblast cells analyzed by SNES. Additional file 4: Figure S2. Processing pipeline for variant detection and annotation. (a) Reads from single-cell exome sequencing are processed by alignment to the human genome, followed by local realignment around indels and removal of PCR duplicates. (b) Error prone reads are filtered by mapping quality, clustering, coverage depth, and the number of variant reads, followed by variant detection using the Unified genotyper to detect SNVs and indels. (c) Multiple databases are integrated for annotation of the variants and to predict the functional impact of the variant on the protein. Additional file 5: Figure S3. Distribution of random FP errors along chromosomes. The frequency of FP errors observed in each single cell are plotted along each chromosome. Frequency counts above four cells were not observed (out of N = 19 cells), and therefore the y-axis scale has shows a limit at four cells. Additional file 6: Figure S4. Allelic dropout bias in single-cell data. Allelic dropout events were calculated from the single-cell exome data and classified into two categories (AB to AA, and AB to BB). The frequency of AB to AA and AB to BB dropout events were calculated and displayed as a stacked histogram. Additional file 7: Figure S5. Distribution of recurrent allelic dropout events along chromosomes. The frequency of allelic dropout errors observed in each single cell are plotted along each chromosome. Many ADO events are recurrent, occurring in multiple single cells.

## References

[1] Ramskold D, Luo S, Wang YC, Li R, Deng Q, Faridani OR (2012). Full-length mRNA-Seq from single-cell levels of RNA and individual circulating tumor cells. Nat Biotechnol..

[2] Tang F, Barbacioru C, Wang Y, Nordman E, Lee C, Xu N (2009). mRNA-Seq whole-transcriptome analysis of a single cell. Nat Methods..

[3] Islam S, Kjallquist U, Moliner A, Zajac P, Fan JB, Lonnerberg P (2011). Characterization of the single-cell transcriptional landscape by highly multiplex RNA-seq. Genome Res..

[4] Hashimshony T, Wagner F, Sher N, Yanai I (2012). CEL-Seq: single-cell RNA-Seq by multiplexed linear amplification. Cell Rep..

[5] Wang J, Fan HC, Behr B, Quake SR (2012). Genome-wide single-cell analysis of recombination activity and de novo mutation rates in human sperm. Cell..

[6] Lohr JG, Adalsteinsson VA, Cibulskis K, Choudhury AD, Rosenberg M, Cruz-Gordillo P (2014). Whole-exome sequencing of circulating tumor cells provides a window into metastatic prostate cancer. Nat Biotechnol..

[7] Navin N, Kendall J, Troge J, Andrews P, Rodgers L, McIndoo J (2011). Tumour evolution inferred by single-cell sequencing. Nature..

[8] Baslan T, Kendall J, Rodgers L, Cox H, Riggs M, Stepansky A (2012). Genome-wide copy number analysis of single cells. Nat Protoc..

[9] Hou Y, Song L, Zhu P, Zhang B, Tao Y, Xu X (2012). Single-cell exome sequencing and monoclonal evolution of a JAK2-negative myeloproliferative neoplasm. Cell..

[10] Zong C, Lu S, Chapman AR, Xie XS (2012). Genome-wide detection of single-nucleotide and copy-number variations of a single human cell. Science..

[11] Wang Y, Waters J, Leung ML, Unruh A, Roh W, Shi X (2014). Clonal evolution in breast cancer revealed by single nucleus genome sequencing. Nature..

[12] Hicks J, Krasnitz A, Lakshmi B, Navin NE, Riggs M, Leibu E (2006). Novel patterns of genome rearrangement and their association with survival in breast cancer. Genome Res..

[13] Beroukhim R, Mermel CH, Porter D, Wei G, Raychaudhuri S, Donovan J (2010). The landscape of somatic copy-number alteration across human cancers. Nature..

[14] Lorenz MO (1905). Methods of measuring the concentration of wealth. J Am Stat Assoc..

[15] Hodges E, Xuan Z, Balija V, Kramer M, Molla MN, Smith SW (2007). Genome-wide in situ exon capture for selective resequencing. Nat Genet..

[16] Cibulskis K, Lawrence MS, Carter SL, Sivachenko A, Jaffe D, Sougnez C (2013). Sensitive detection of somatic point mutations in impure and heterogeneous cancer samples. Nat Biotechnol..

[17] McKenna A, Hanna M, Banks E, Sivachenko A, Cibulskis K, Kernytsky A (2010). The Genome Analysis Toolkit: a MapReduce framework for analyzing next-generation DNA sequencing data. Genome Res..

[18] Dean FB, Hosono S, Fang L, Wu X, Faruqi AF, Bray-Ward P (2002). Comprehensive human genome amplification using multiple displacement amplification. Proc Natl Acad Sci U S A..

[19] Lasken RS (2007). Single-cell genomic sequencing using Multiple Displacement Amplification. Curr Opin Microbiol..

[20] Xu X, Hou Y, Yin X, Bao L, Tang A, Song L (2012). Single-cell exome sequencing reveals single-nucleotide mutation characteristics of a kidney tumor. Cell..

[21] de Vega M, Lazaro JM, Mencia M, Blanco L, Salas M (2010). Improvement of phi29 DNA polymerase amplification performance by fusion of DNA binding motifs. Proc Natl Acad Sci U S A..

[22] Adey A, Morrison HG (2010). Asan, Xun X, Kitzman JO, Turner EH, et al. Rapid, low-input, low-bias construction of shotgun fragment libraries by high-density in vitro transposition. Genome Biol..

[23] Wang Q, Gu L, Adey A, Radlwimmer B, Wang W, Hovestadt V (2013). Tagmentation-based whole-genome bisulfite sequencing. Nat Protoc..

[24] Picelli S, Bjorklund AK, Reinius B, Sagasser S, Winberg G, Sandberg R (2014). Tn5 transposase and tagmentation procedures for massively scaled sequencing projects. Genome Res..

[25] Marine R, Polson SW, Ravel J, Hatfull G, Russell D, Sullivan M (2011). Evaluation of a transposase protocol for rapid generation of shotgun high-throughput sequencing libraries from nanogram quantities of DNA. Appl Environ Microbiol..

[26] de Bourcy CF, De Vlaminck I, Kanbar JN, Wang J, Gawad C, Quake SR (2014). A quantitative comparison of single-cell whole genome amplification methods. PLoS One..

[27] Gole J, Gore A, Richards A, Chiu YJ, Fung HL, Bushman D (2013). Massively parallel polymerase cloning and genome sequencing of single cells using nanoliter microwells. Nat Biotechnol..

[28] Ni X, Zhuo M, Su Z, Duan J, Gao Y, Wang Z (2013). Reproducible copy number variation patterns among single circulating tumor cells of lung cancer patients. Proc Natl Acad Sci U S A..

[29] Crasta K, Ganem NJ, Dagher R, Lantermann AB, Ivanova EV, Pan Y (2012). DNA breaks and chromosome pulverization from errors in mitosis. Nature..

[30] Langmead B, Trapnell C, Pop M, Salzberg SL (2009). Ultrafast and memory-efficient alignment of short DNA sequences to the human genome. Genome Biol..

[31] Li H, Handsaker B, Wysoker A, Fennell T, Ruan J, Homer N (2009). The Sequence Alignment/Map format and SAMtools. Bioinformatics..

[32] Picard Tools. [http://broadinstitute.github.io/picard/].

[33] Quinlan AR, Hall IM (2010). BEDTools: a flexible suite of utilities for comparing genomic features. Bioinformatics..

