# Supplementary figures and images for: SNES: single nucleus exome sequencing
Source: Genome Biol. 2015 Mar 25;16(1):55. doi: 10.1186/s13059-015-0616-2 (PMC4373516; doi:10.1186/s13059-015-0616-2)

Cell Counts

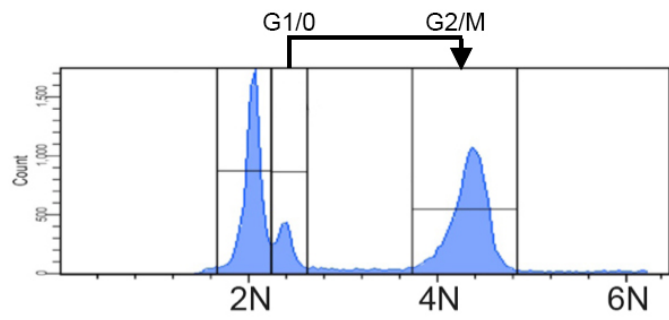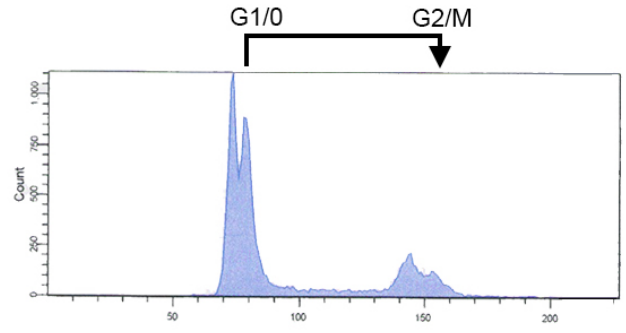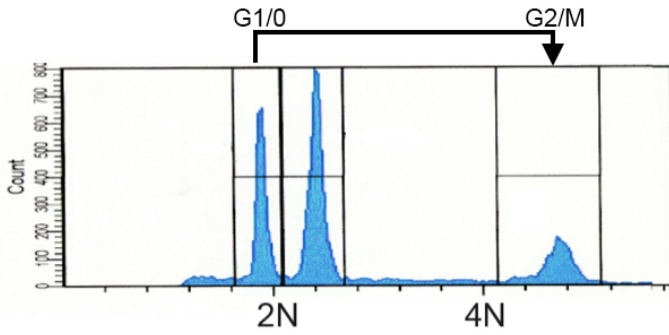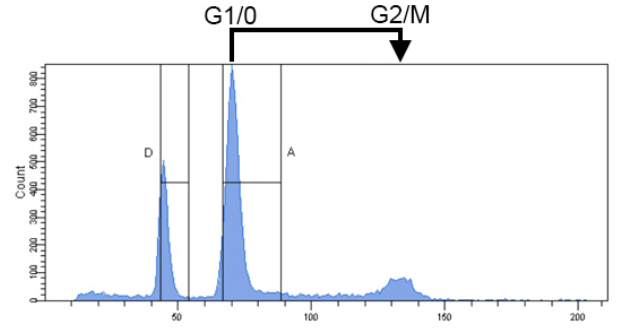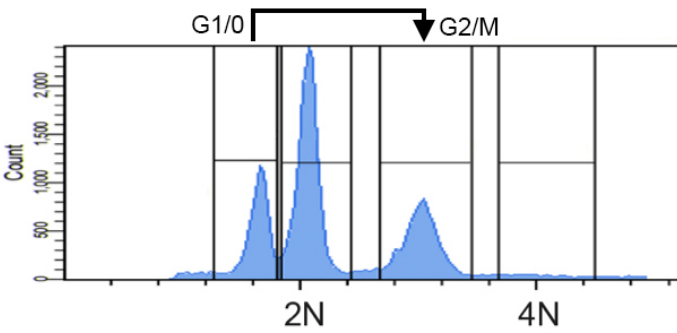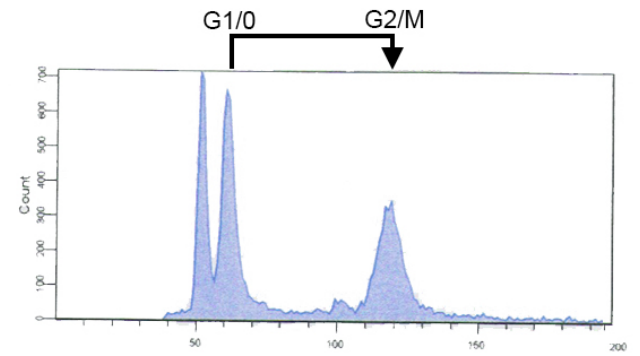

DNA Ploidy

Supplement: Additional file 1: Figure S1. — G1/0 and G2/M ploidy distributions in aneuploid breast tumors. Nuclei were prepared from frozen breast tumors and stained with DAPI. The nuclear suspensions were analyzed by cytometric analysis showing the distributions of total DNA content. The aneuploid distributions are highlighted for G1/0 and the corresponding G2/M populations in each frozen tumor sample. [file 13059_2015_616_MOESM1_ESM.pdf]

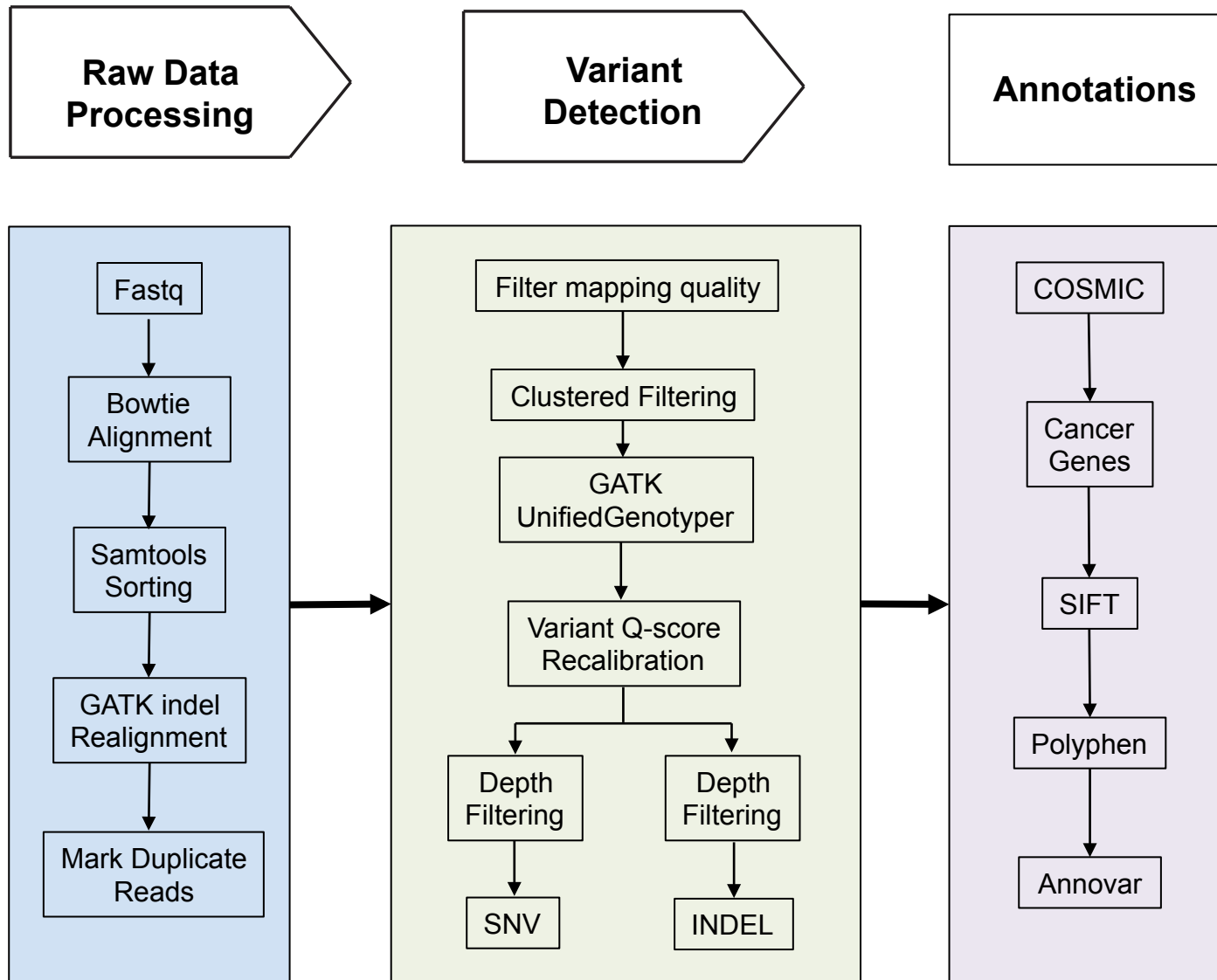

Supplement: Additional file 4: Figure S2. — Processing pipeline for variant detection and annotation. (a) Reads from single-cell exome sequencing are processed by alignment to the human genome, followed by local realignment around indels and removal of PCR duplicates. (b) Error prone reads are filtered by mapping quality, clustering, coverage depth, and the number of variant reads, followed by variant detection using the Unified genotyper to detect SNVs and indels. (c) Multiple databases are integrated for annotation of the variants and to predict the functional impact of the variant on the protein. [file 13059_2015_616_MOESM4_ESM.pdf]

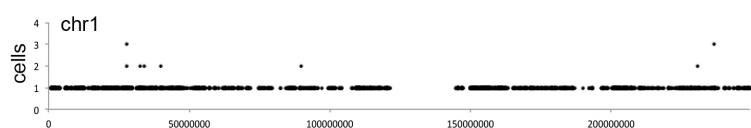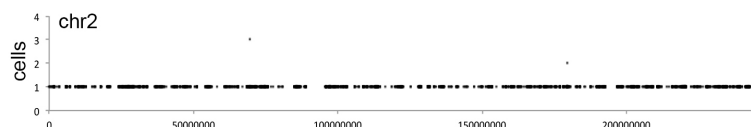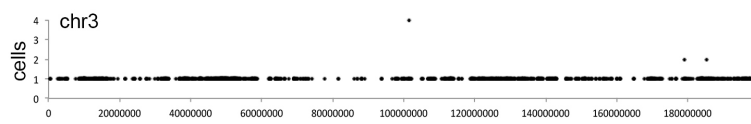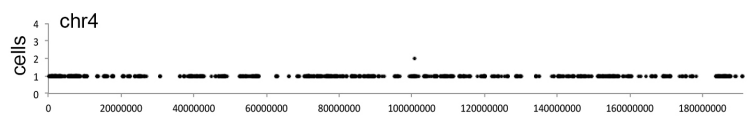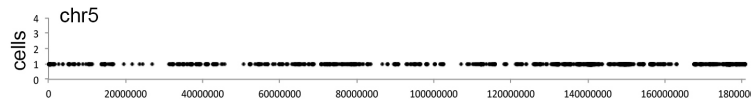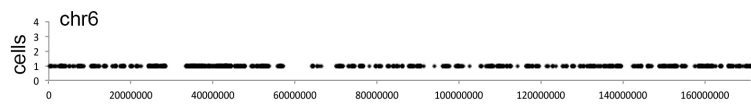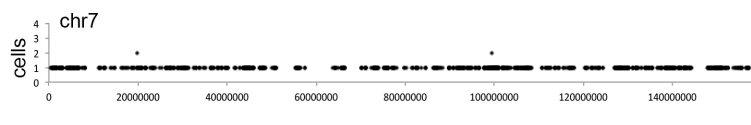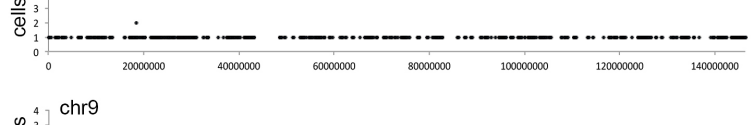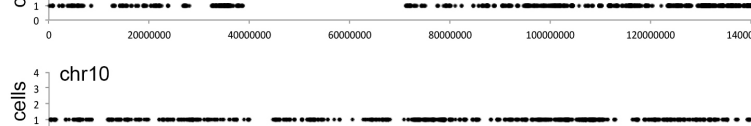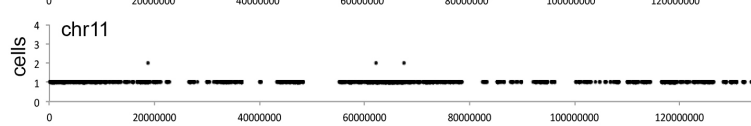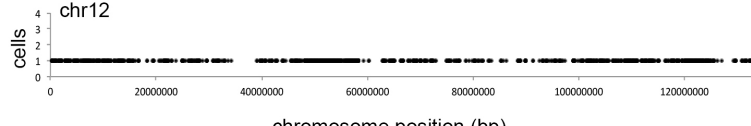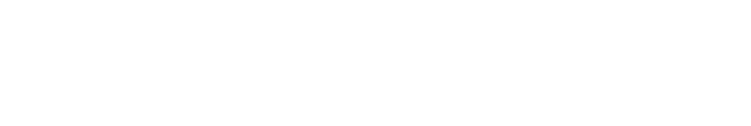

chromosome position (bp)

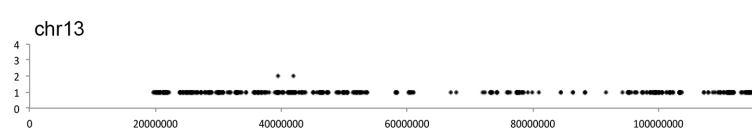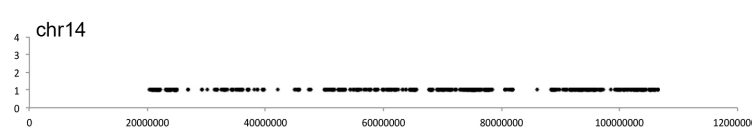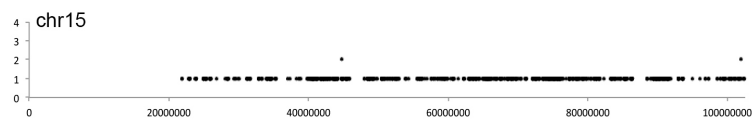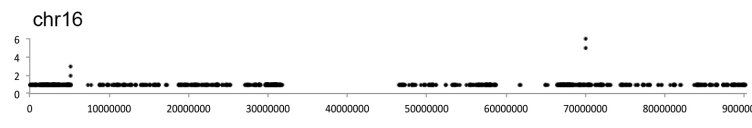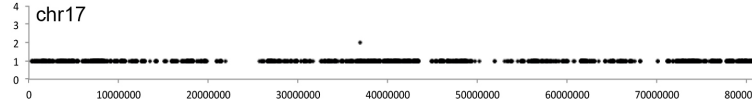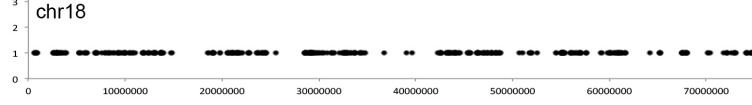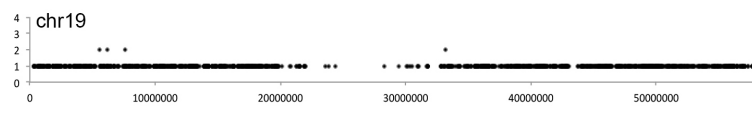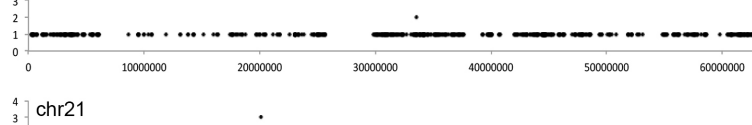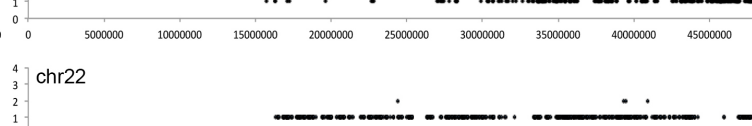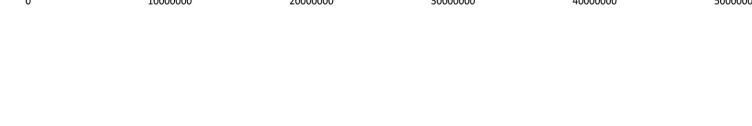

chromosome position (bp)

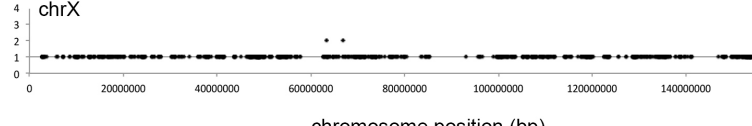

Supplement: Additional file 5: Figure S3. — Distribution of random FP errors along chromosomes. The frequency of FP errors observed in each single cell are plotted along each chromosome. Frequency counts above four cells were not observed (out of N = 19 cells), and therefore the y-axis scale has shows a limit at four cells. [file 13059_2015_616_MOESM5_ESM.pdf]

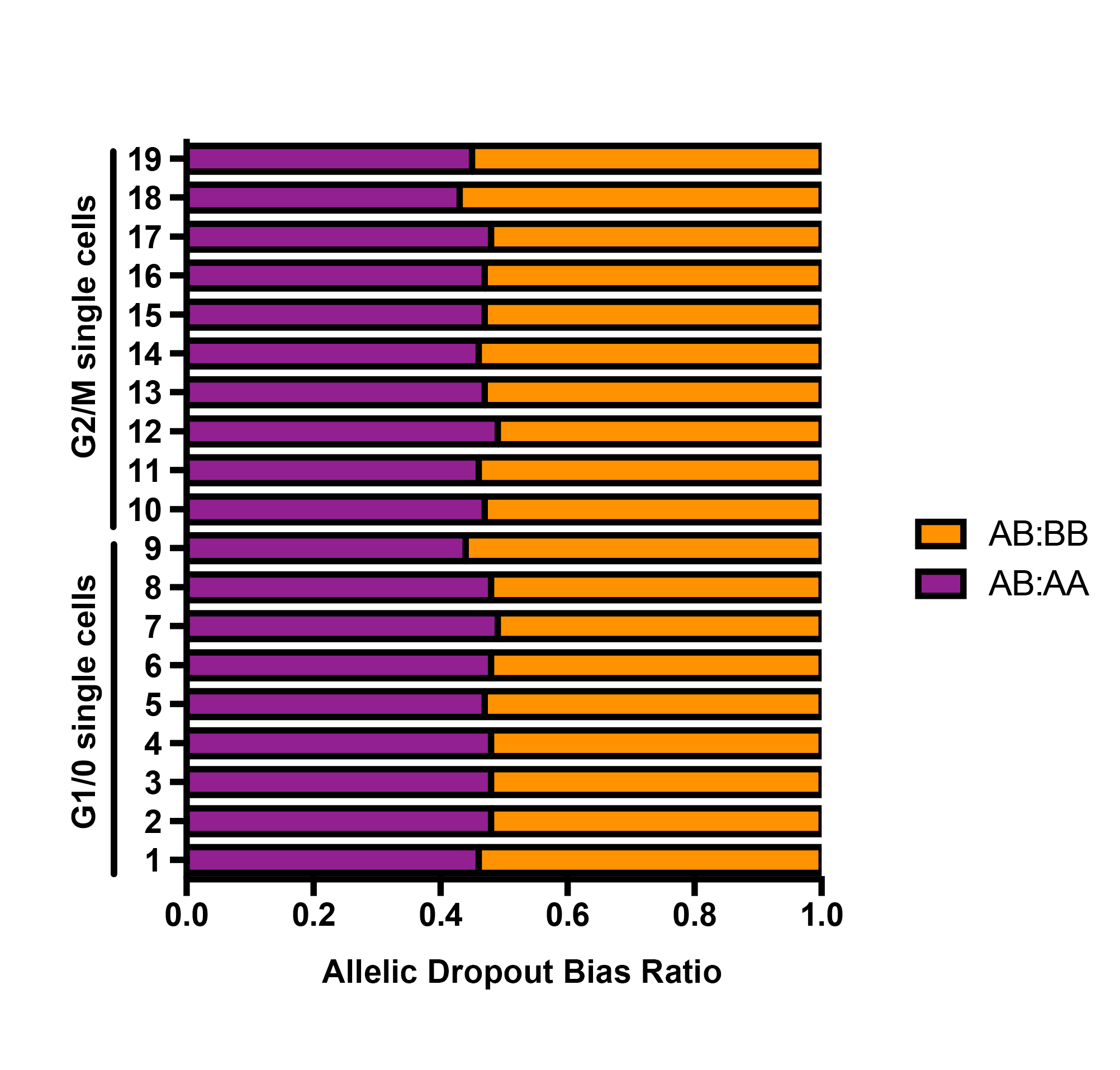

Supplement: Additional file 6: Figure S4. — Allelic dropout bias in single-cell data. Allelic dropout events were calculated from the single-cell exome data and classified into two categories (AB to AA, and AB to BB). The frequency of AB to AA and AB to BB dropout events were calculated and displayed as a stacked histogram. [file 13059_2015_616_MOESM6_ESM.jpeg]
