# Supplementary material for: SNES: single nucleus exome sequencing
Source: Genome Biol. 2015 Mar 25;16(1):55. doi: 10.1186/s13059-015-0616-2 (PMC4373516; doi:10.1186/s13059-015-0616-2)
Supplement: Additional file 2: Table S1. — Chromosome specific qPCR primers. Summary table of the chromosome-specific primer panel DNA sequences that are used to perform quality control of the single cell WGA reactions. [file 13059_2015_616_MOESM2_ESM.pdf]

Additional file 2: Table S1 - Chromosome Specific qPCR Primers

Name	Chrom	Orientation	Primer Sequence
Chr1F	1	forward	TCCAAGCTCCAGTTCCAGAT
Chr1R	1	reverse	TGCACTGAGACCTTCACAGG
Chr2F	2	forward	AGCGGGAGGGACTATTTCAC
Chr2R	2	reverse	GGATCGTTCAAAGGGAACTG
Chr3F	3	forward	CCCTTGTACTGGCTCGTGTT
Chr3R	3	reverse	CTTGCACATGAAGGTCTGGA
Chr4F	4	forward	GAGCATCTCTTGGCTCTGCT
Chr4R	4	reverse	TTGGGAAAGCACAGATCCTT
Chr5F	5	forward	TTGCAGCTTTCCATTACGTG
Chr5R	5	reverse	CCTTTTATGCCTCCAGCATC
Chr6F	6	forward	GAGGAGGGCAAGGAGAGAGT
Chr6R	6	reverse	ACCCTCCAGTGTGCAAAAAC
Chr7F	7	forward	CTTCCTGCCATTCCACAAGT
Chr7R	7	reverse	CCCACTTTCATGCCTCTGAT
Chr8F	8	forward	CTTCCCTGCCTTGCTCTCTA
Chr8R	8	reverse	CGGGACATTTCAGCAATCTT
Chr9F	9	forward	CTGTGGAGCAGCTGTTTCTG
Chr9R	9	reverse	GAATTCACAAAGCCCCAAGA
Chr10F	10	forward	CCCCTCATTCAAATCAGCAT
Chr10R	10	reverse	CAGGCAAAAGCTGGAGTTTC
Chr11F	11	forward	AGCATCATCCAGCCCATTAC
Chr11R	11	reverse	AAATCCCTGCAGAGCAGTGT
Chr12F	12	forward	ATCATGGAAATGCAGCCTCT
Chr12R	12	reverse	AGAACCCAGCTGGAATGATG
Chr13F	13	forward	TGTTTCATGGAGTCCTGCTG
Chr13R	13	reverse	GGAGGCAAGAACCAAACAAA
Chr14F	14	forward	AGCCAAGACGTACCCTCTCA
Chr14R	14	reverse	TGCTTTACACCAATCCCACA
Chr15F	15	forward	TCAGCATGGGTTATGGGTTT
Chr15R	15	reverse	CCCAGATGATGGAGAGGAAA
Chr16F	16	forward	GCCTGTGTTTGCTGATGAAA
Chr16R	16	reverse	GGGCAACGACCGTACTTAAA
Chr17F	17	forward	TCCTGGGCTAGCCTTTTACA
Chr17R	17	reverse	ATCGCTTGAGCACTGAAGGT
Chr18F	18	forward	AGACGAGCCTTTCTCTGTCG
Chr18R	18	reverse	TCGAGACCATCCCCACTAAC
Chr19F	19	forward	TACTCAAAGCTGGCAGCAGA
Chr19R	19	reverse	GAGCATGCCCAGGATACCTA
Chr20F	20	forward	CACCAGGGTCTTGATGGAGT
Chr20R	20	reverse	AGCTCTGGGATCTGTGATGG
Chr21F	21	forward	TGGACAAATAAAGGCAATGG
Chr21R	21	reverse	TCAGGCAACTTCTGGATGAA
Chr22F	22	forward	CTAGGATCCCGTGAAGGTCA
Chr22R	22	reverse	AGGTAAGGGGACTCCTTGGT
