# Supplementary material for: SNES: single nucleus exome sequencing
Source: Genome Biol. 2015 Mar 25;16(1):55. doi: 10.1186/s13059-015-0616-2 (PMC4373516; doi:10.1186/s13059-015-0616-2)
Supplement: Additional file 3: Table S2. — Single nuclei exome sequencing metrics. Summary table for the coverage and sequencing metrics from single-cell exome sequencing of the G1/0 and G2/M fibroblast cells analyzed by SNES. [file 13059_2015_616_MOESM3_ESM.pdf]

Additional file 3: Table S2 - Single Nuclei Exome Sequencing Metrics

Cell ID	Sample ID	cell type	Gate	Coverage Depth	Coverage Breadth	on-target	off-target	Mapped reads	Duplicates	Duplicate Fraction
cell1	SKN2-3	fibroblast	G1/0	92	0.9656	0.694434	0.305566	75807655	4499437	0.0593533
cell2	SKN2-1	fibroblast	G1/0	86	0.9597	0.725463	0.274537	116959349	6840425	0.0584855
cell3	SKN2-8	fibroblast	G1/0	63	0.8734	0.698274	0.301726	83050018	4272458	0.0514444
cell4	SKN2-9	fibroblast	G1/0	61	0.9199	0.717339	0.282661	130394134	7879773	0.0604304
cell5	SKN2-2	fibroblast	G1/0	60	0.9265	0.683081	0.316919	82702966	4637801	0.0560778
cell6	SKN2-6	fibroblast	G1/0	58	0.881	0.692569	0.307431	79167508	4133589	0.0522132
cell7	SKN2-5	fibroblast	G1/0	57	0.808	0.710105	0.289895	81729806	4740215	0.0579986
cell8	SKN2-7	fibroblast	G1/0	57	0.8964	0.697603	0.302397	88341020	4853585	0.0549415
cell9	SKN2-4	fibroblast	G1/0	54	0.8335	0.683449	0.316551	78950029	4716958	0.0597461
cell10	SKN2-1	fibroblast	G2/M	83	0.9768	0.717238	0.282762	113932151	6674671	0.0585846
cell11	SKN2-4	fibroblast	G2/M	81	0.9758	0.706091	0.293909	79911960	4682799	0.0585995
cell12	SKN2-3	fibroblast	G2/M	76	0.9729	0.707748	0.292252	75800320	3599405	0.0474854
cell13	SKN2-7	fibroblast	G2/M	67	0.956	0.681542	0.318458	80648480	3964360	0.049156
cell14	SKN2-2	fibroblast	G2/M	60	0.9709	0.688752	0.311248	82272279	4248310	0.0516372
cell15	SKN2-8	fibroblast	G2/M	60	0.9571	0.703352	0.296648	66219143	3081922	0.0465413
cell16	SKN2-9	fibroblast	G2/M	58	0.9537	0.692903	0.307097	112665033	6590337	0.058495
cell17	SKN2-6	fibroblast	G2/M	56	0.9475	0.678661	0.321339	92501465	4228379	0.0457115
cell18	SKN2-5	fibroblast	G2/M	54	0.952	0.682259	0.317741	94910923	4911964	0.0517534
cell19	SKN2-10	fibroblast	G2/M	47	0.9315	0.68334	0.31666	103888986	5685038	0.0547222
