# Supplementary material for: SNES: single nucleus exome sequencing
Source: Genome Biol. 2015 Mar 25;16(1):55. doi: 10.1186/s13059-015-0616-2 (PMC4373516; doi:10.1186/s13059-015-0616-2)
Supplement: Additional file 7: Figure S5. — Distribution of recurrent allelic dropout events along chromosomes. The frequency of allelic dropout errors observed in each single cell are plotted along each chromosome. Many ADO events are recurrent, occurring in multiple single cells. [file 13059_2015_616_MOESM7_ESM.pdf]

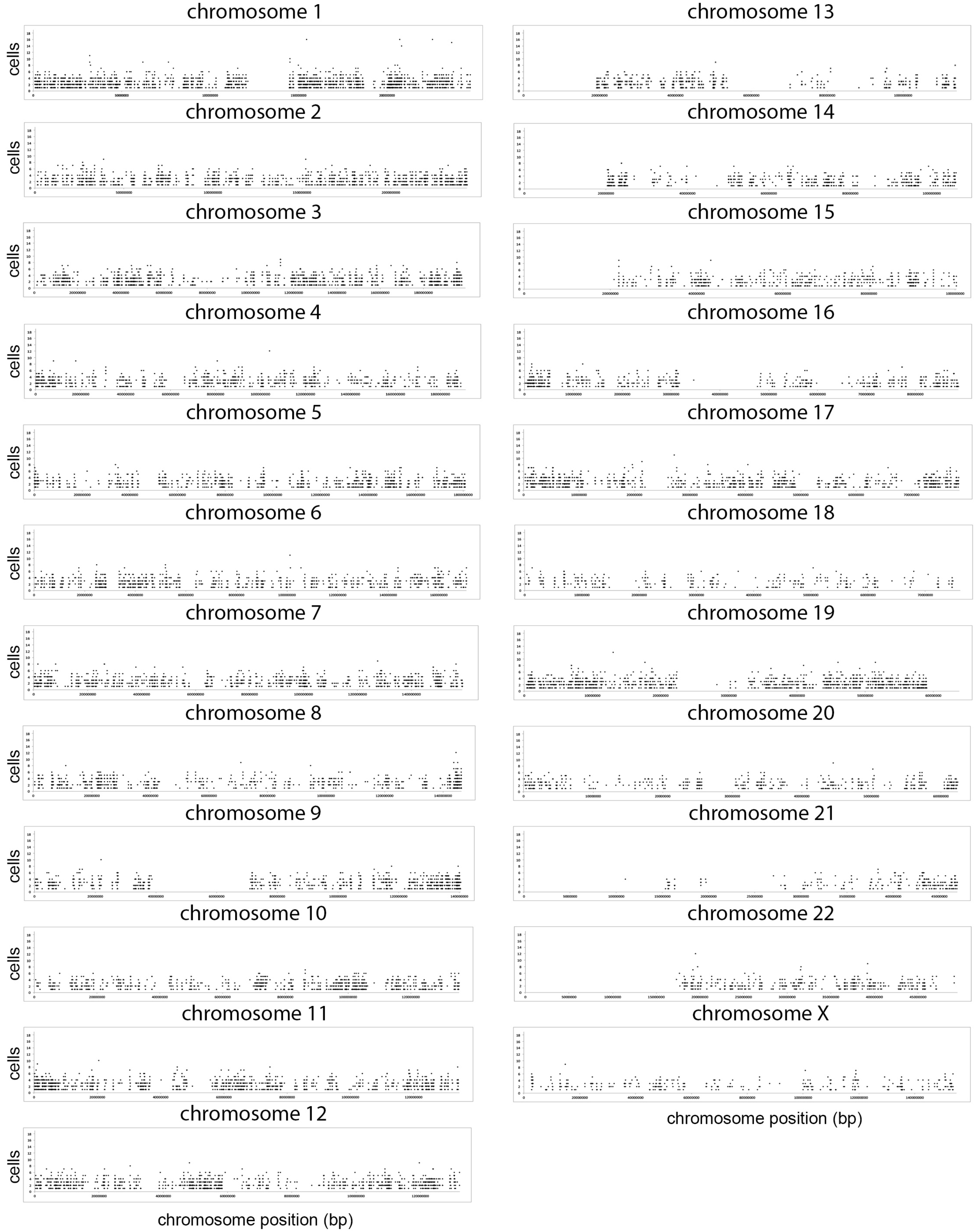
chromosome 1
chromosome 2
chromosome 3
chromosome 4
chromosome 5
chromosome 6
chromosome 7
chromosome 8
chromosome 9
chromosome 10
chromosome 11
chromosome 12
chromosome 13
chromosome 14
chromosome 15
chromosome 16
chromosome 17
chromosome 18
chromosome 19
chromosome 20
chromosome 21
chromosome 22
chromosome X
cells
chromosome position (bp)
